# Health-Related Quality of Life and Health Service Utilization in Chinese Rural-to-Urban Migrant Workers

**DOI:** 10.3390/ijerph120202205

**Published:** 2015-02-16

**Authors:** Chu-Hong Lu, Zhong-Cheng Luo, Jia-Ji Wang, Jian-Hu Zhong, Pei-Xi Wang

**Affiliations:** 1 Department of Preventive Medicine, School of Public Health, Guangzhou Medical University, Guangzhou 510182, China; E-Mails: chuhong688@163.com (C.-H.L.); wjiaji@163.com (J.-J.W.); 2 Institute of Public Health, School of Nursing, Henan University, Kaifeng 475004, China; 3 Ministry of Education-Shanghai Key Laboratory of Children’s Environmental Health, Xin Hua Hospital, Shanghai Jiao-Tong University School of Medicine, Shanghai 200092, China; E-Mail: zhongcheng_luo3@163.com; 4 Community Health Services Center of Liaobu, Dongguan 523401, China; E-Mail: edwardzjh@163.com

**Keywords:** health-related quality of life (HRQOL), health service utilization, migrant workers

## Abstract

*Objectives*: The number of rural-to-urban migrant workers has been increasing rapidly in China over recent decades, but there is a scarcity of data on health-related quality of life (HRQOL) and health service utilization among Chinese rural-to-urban migrant workers in comparison to local urban residents. We aimed to address this question. *Methods*: This was a cross-sectional study of 2315 rural-to-urban migrant workers and 2347 local urban residents in the Shenzhen-Dongguan economic zone (China) in 2013. Outcomes included HRQOL (measured by Health Survey Short Form 36) and health service utilization (self-reported). *Results*: Compared to local urban residents, rural-to-urban migrant workers had lower scores in all domains of HRQOL, and were more likely to report chronic illnesses (9.2% *vs.* 6.0%, adjusted OR = 1.62, 95% CI 1.28–2.04) and recent two-week morbidity (21.3% *vs.* 5.0%, adjusted OR = 5.41, 95% CI 4.26–6.88). Among individuals who reported sickness in the recent two weeks, migrant workers were much less likely to see a doctor (32.7% *vs.* 66.7%, adjusted OR = 0.21, 95% CI 0.13–0.36). *Conclusions*: Chinese rural-to-urban migrant workers have lower HRQOL, much more frequent morbidity, but are also much less likely to see a doctor in times of sickness as compared to local urban residents, indicating the existence of significant unmet medical care needs in this population.

## 1. Introduction

The number of rural-to-urban migrants has been increasing dramatically in China over the last three decades [1,2], a result of the country’s rapid economic development and urbanization. From just 70 million in 1993, China’s rural-to-urban migrant population quadrupled to 268.9 million in 2013 [3,4,5]. Migrants have made a major contribution to China’s industrial development and economic growth, but they face a variety of public health and social problems [6]. The Chinese household registration system (*fukou*) classifies individuals as rural and urban citizens by law, and rural-to-urban migrants are classified as temporary urban residents regardless of their length of stay in cities. Employment opportunities and social welfare benefits differ according to the household registration status. Many social welfare benefits in urban areas are only available to registered urban citizens, but not to registered rural citizens who work and live in urban areas [1,2,7,8]. The household registration system serves to reduce population pressure in China's crowded cities by preventing large-scale migration of the population to cities on a permanent basis [9]. Migrants experience a variety of inequalities including long working hours, insecure employment, overcrowded and insalubrious living conditions [6,10,11]. They are generally marginalized in urban areas [12].

The health status of migrant workers is an increasingly recognized public health concern in China. Concerns for migrants' health have been centered on the consequences of dangerous working conditions with high occupational health risks, poor living conditions with crowded housing, and limited access to clean water and sanitation [13,14]. There is a scarcity of data on health-related quality of life (HRQOL) and health service utilization among rural-to-unban migrant workers as compared with local residents in China. HRQOL is an essential aspect of human health embedded in a physical, mental and social context [15]. We are aware of only a few limited studies on HRQOL among rural-to-urban migrants. Zhu and colleagues reported that HRQOL in rural-urban female migrant workers was lower as compared to Chinese female norms [10]. Liu *et al*. showed that the HRQOL among migrant workers was significantly lower than among governmental civil employees [16]. However, the most appropriate comparison group for such a study would be local residents of the same city, rather than the general Chinese population or governmental employees. To the best of our knowledge, there remains a lack of data comparing HRQOL and health service utilization among Chinese rural-to-unban migrant workers and local residents living in the same region. To address this gap the goal of this study was to evaluate HRQOL and health service utilization among migrant workers as compared to local residents in the Shenzhen-Dongguan economic zone—a leading modern light industrial area in China.

## 2. Methods

### 2.1. Study Design and Participants

This was a cross-sectional study performed between April and May 2013. Rural-to-urban migrant light-duty manual workers (18–59 years of age) were sampled from three light-industry factories without strong occupational health risk hazards in Shenzhen-Dongguan economic zone in China—a textile manufacturer, a furniture assembler and a camera assembler. The migrant workers usually worked about 10 hours per day during weekdays, and lived in the factory area. Most of them had primary school or junior high school education. Migrant workers between 18 and 59 years of age in the three factories were sampled by a cluster randomization method. We randomly sampled 20 of 61 workshops in these factories (about 1/3 of the total). Migrants were defined as individuals who came from rural areas and had worked in the urban area for more than three months. There were a total of 2460 rural-to-urban migrant workers in the 20 workshops; 145 of them were under the age of 18 and thus ineligible. All eligible subjects (n = 2315; including 987 males and 1328 females) in the sampled workshops were recruited via face-to-face interviews by trained study personnel upon informed consent; none refused to participate.

For comparisons, local residents between 18 and 59 years of age were randomly sampled in the general population through the city’s household registration system. For most families, there are three or four family members, including a married couple and 1–2 unmarried children. Only one participant within each family, usually a household host (the father or mother if available), was recruited for the interview to ensure independence of study subjects. We interviewed the first host we met in a family. They were identified consecutively and interviewed at home by trained study personnel. If a potential participant refused to participate or did not respond to the invitation, a next-door available neighbor was invited. We recruited 2347 local residents including 1040 males and 1307 females (similar to the sample size in the migrant workers study group) as the comparison group. The study was approved by the Research Ethics Board of Guangzhou Medical University. Written informed consent was obtained from all study participants.

### 2.2. Procedures

Data were collected via face-to-face interviews performed by trained study personnel. Well-trained medical student interviewers from Guangzhou Medical University collected research data using structured study questionnaires. The interviewers received training and engaged in group discussions and simulated interviews to improve their interview skills and standardize the data collection procedures.

### 2.3. Basic Questionnaire 

The basic questionnaire covered information items on demographic characteristics and health service utilization. Demographic variables included age, sex, marital status, income, medical insurance and education. Health service utilization variables included chronic diseases, recent two-week morbidity, recent two-week physician visits, conditions requiring hospitalization, and annual hospitalization. Chronic diseases in the questionnaire included hypertension, diabetes, and “other” chronic diseases. “Recent two-week morbidity” was obtained in the question “Have you been ill over the past 2 weeks?”. “Recent two-week physician visit” was obtained in the question “In the past two weeks, have you ever visited a doctor when you were sick?”. “Conditions requiring hospitalization" was obtained in the question “In the last year, have you had conditions where hospitalization was recommended by a doctor?”. “Annual hospitalization” was obtained in the question “In the last year, have you been hospitalized?”.

### 2.4. SF-36 Questionnaire

The participants were asked to evaluate their HRQOL using the Chinese version of the Health Survey Short Form (SF-36), which was translated from the standard English version of SF-36. The SF-36 questionnaire showed satisfactory construction and clinical validity and internal consistency for measuring HRQOL [17,18,19]. It comprises 36 questions about various aspects of health covering eight domains: four in the area of physical health (PCS, Physical Component Summary) including physical functioning (PF, limitations in physical activities such as bathing or dressing because of health problems, a total of 10 items), physical roles (RP, limitations in usual role activities such as work or other daily activities because of physical health problems, a total of four items), bodily pain (BP, how severe and limiting is pain, a total of two items) and general health (GH, general health perceptions, a total of five items); four in the area of mental health (MCS, Mental Component Summary) including vitality (VT, feeling full of energy *vs.* feeling tired and worn out, a total of four items), social functioning (SF, limitations in social activities because of physical or emotional problems, a total of 2 items), role-emotional (RE, limitations in usual role activities such as work or other daily activities because of emotional problems, a total of three items) and mental health (MH, feeling nervous and depressed *vs.* peaceful, happy and calm, a total of five items). Both physical and mental health scores have been empirically validated [20]. The SF-36 items were scored according to published scoring procedures, and each domain was scored in the range of 0–100. Higher scores indicate better HRQOL. The MCS and PCS scores were calculated according to Lam *et al.* [21]

### 2.5. Statistical Analysis

Statistical analyses were conducted using Statistical Package for Social Sciences (SPSS), version 13.0 (SPSS, Inc, Chicago, IL, USA). For two-group comparisons, we used independent sample t test or analysis of variance for continuous variables, and Chi square test for categorical variables. Independent-samples T test was used to compare the means of HRQOL between two groups. Logistic regression was used to evaluate factors associated with dichotomous outcomes (e.g. recent two-week physician visit, yes/no) comparing migrant workers to local urban residents adjusted for co-variables. *p* values < 0.05 were considered statistically significant.

## 3. Results

The characteristics of study participants are presented in Table 1. As compared to local residents, rural-to-urban migrant workers were less likely to be married (*p* < 0.001) or to have medical insurance (*p* = 0.003), but more likely to have lower education levels (*p* < 0.001), and had lower income (*p* < 0.001). 

**Table 1:** Characteristics of rural-to-urban migrant workers and local urban residents in the study population, Shenzhen-Dongguan economic zone, China.

Variable	Rural–to-urban migrant workers (n = 2315)	Local urban residents (n = 2347)	*p* ^a^
Age (years)	33.1 ± 8.7	33. 6 ± 8.2	0.052
Sex (male/female)	987/1328	1040/1307	0.248
Marital status, married (yes)	1657 (71.6%)	2065 (88.0%)	<0.001
Average monthly income	2992.6 ± 731.3	4814.3 ± 5839.6	<0.001
Medical insurance (yes)	1986 (85.8%)	2081 (88.7%)	0.003
Education			<0.001
Primary school or lower	393 (17.0%)	174 (7.4%)	
Junior high school	1367 (59.0%)	741 (31.6%)	
High school or above	555 (24.0%）	1432 (61.0%)	
Chronic diseases			<0.001
0	2101 (90.8%）	2207 (94.0%）	
1 or more	214 (9.2%）	140 (6.0%）	
Morbidity, recent 2 weeks			<0.001
0	1822 (78.7%）	2230 (95.0%）	
1 or more	493 (21.3%）	117 (5.0%）	
Physician visits, recent 2 weeks			<0.001
0	2152 (93.0%）	2269 (96.7%）	
1 or more	163 (7.0%）	78 (3.3%）	
Conditions requiring hospitalization			<0.001
0	2178 (94.1%)	2120 (90.3%)	
1 or more	137 (5.9%)	227 (9.7%)	
Hospitalizations, last year			<0.001
0	2191 (94.6%）	2128 (90.7%）	
1 or more	124 (5.4%）	219 (9.3%）	

Notes: Data presented are mean± SD or n (%). ^a^ *p* values in t-test (age, average monthly income) or Chi-square test (other variables) for differences between the two groups.

Rural-to-urban migrant workers were also more likely to have reported chronic diseases (9.2% *vs.* 6.0%), recent two-week morbidity (21.3% *vs.* 5.0%) or physician visits (7.0% *vs.* 3.3%) than local residents. However, conditions requiring hospitalization (5.9% *vs.* 9.7%) and hospitalizations in the last year (5.4% *vs.* 9.3%) were less frequent in migrant workers than in local residents. Age and sex did not differ significantly between the two groups.

Data on HRQOL are presented in Table 2. The scores in different HRQOL domains varied from 70.6 (SD = 18.2) to 93.8 (11.4) in rural-to-urban migrant workers, and from 73.3 (15.3) to 97.6 (7.5) in local residents. The overall mean PCS and MCS scores were 47.7 (9.4) and 48.2 (10.7) in migrant workers, *vs.* 52.2 (6.7) and 51.8 (7.7) in local residents, respectively. Compared to local residents, rural-to-urban migrant workers showed significantly lower scores in all HRQOL domains and overall mean scores of PCS and MCS. The differences were significant (*p* < 0.001) even after adjusting for age, sex, marital status, medical insurance and education.

**Table 2:** Health related quality of life (HRQOL) in rural-to-urban migrant workers *versus* local residents.

HRQOL Domain	Rural-to-Urban Migrant Workers	Local Urban Residents	*p* ^a^	*p* ^b^
PF	93.8 ± 11.4	97.6 ± 7.5	<0.001	<0.001
RP	85.0 ± 26.4	96.8 ± 14.0	<0.001	<0.001
BP	85.1 ± 17.1	94.6 ± 11.8	<0.001	<0.001
GH	71.4 ± 18.3	73.3 ± 15.3	<0.001	0.004
VT	70.6 ± 18.2	76.6 ± 13.5	<0.001	<0.001
SF	89.1 ± 15.4	93.4 ± 11.7	<0.001	<0.001
RE	81.6 ± 30.1	95.3 ± 18.3	<0.001	<0.001
MH	77.8 ± 15.6	80.3 ± 12.7	<0.001	<0.001
PCS	47.7 ± 9.4	52.2 ± 6.7	<0.001	<0.001
MCS	48.2 ± 10.7	51.8 ± 7.7	<0.001	<0.001

Notes: Data presented are mean ± SD. PF = Physical functioning; RP = Role physical; BP = Bodily pain; GH = General health; VT = Vitality; SF = Social functioning; RE = Role-emotional; MH = Mental health. ^a^ Crude P values in t tests for differences between the two groups. ^b^ Adjusted P values for differences between the two groups controlling for age, sex, marital status, average monthly income, medical insurance and education. Adjusted for age, sex, marital status, medical insurance and education, rural-to-urban migrant workers remained significantly more likely to report chronic diseases (OR = 1.62, *p* < 0.001), recent two-week morbidity (OR = 5.41, *p* < 0.001) or physician visit (OR = 2.34, *p* < 0.001), but less likely to report conditions requiring hospitalization(OR = 0.71, *p* = 0.007) or actual hospitalization in the last year (OR = 0.67, *p* = 0.003) as compared to local urban residents (Table 3).

**Table 3:** Odds ratios of self-reported morbidity and health service utilization comparing Chinese rural-to-urban migrant workers *vs.* local urban residents.

Outcome	Crude OR（95%CI）	*p* ^a^	Adjusted OR （95%CI）	*p* ^b^
Chronic diseases	1.50 (1.23–1.83)	<0.001	1.62 (1.28–2.04)	<0.001
Morbidity, recent 2-weeks	5.16（4.18–6.37）	<0.001	5.41 (4.26–6.88)	<0.001
Physician visits, recent 2-weeks	2.20（1.67–2.90）	<0.001	2.34 (1.72–3.20)	<0.001
Conditions requiring hospitalization	0.59（0.47–0.73）	<0.001	0.71 (0.55–0.91)	0.007
Hospitalizations, last year	0.55（0.44–0.69）	<0.001	0.67 (0.52–0.88)	0.003

Notes: ^a^ Crude *p* values for differences between the two groups. ^b^ Adjusted *p* values for differences between the two groups controlling for age, sex, marital status, average monthly income, medical insurance and education.

In a subgroup analysis of subjects who reported any sickness in recent two weeks (n = 610), migrant workers were much less likely to see a doctor than local urban residents [32.7% (161 of 493) *vs.* 66.7% (78 of 117), adjusted OR = 0.21, 95% CI 0.13–0.36, *p* < 0.001].

## 4. Discussion

### 4.1. Main Findings

To the best of our knowledge, this is the first study on HRQOL and health service utilization comparing Chinese rural-to-unban migrant workers to local urban residents living in the same region. Our study revealed that Chinese rural-to-urban migrant workers tend to have lower HRQOL and higher morbidity, but much lower likelihood of seeing a doctor at times of illness than local urban residents.

### 4.2. Health-Related Quality of Life

HRQOL reflects the perception of the quality of an individual’s physical and mental health, and is closely related to self-reported chronic disease and behavioral risk factors [22]. Migrant workers are a vulnerable population group in urban areas. These migrant workers have lower education levels, lower income, heavier work-loads, longer working hours, and live in more crowded housing compared to local residents. Our study showed that migrant workers experienced lower quality of life compared to local residents. Our findings are consistent with those of Zhu’s and Liu’s studies indicating lower HRQOL in rural-to-urban migrant workers [10,16], but in this case using a more appropriate comparison group. We compared migrant workers to local residents, rather than to the general HRQOL norm for Chinese females in Zhu’s study, or to governmental employees in Liu’s study. The lower HRQOL in migrant workers may be explained by poor social support, living and work conditions [6,10,16]. Two-week morbidity rate is a strong predictor of HRQOL [10]. Chronic diseases are associated with lower HRQOL [23,24]. In our study, migrants were more likely to have reported recent two-week morbidity or chronic diseases than local urban residents. This may partly explain their lower HRQOL compared to local residents. In contrast, the study of Hesketh *et al*. reported that migrants had better self-rated health as compared to local urban residents [11]. The reason may be that in Hesketh’s study [11], urban residents (the control group) were drawn from the same work units as the migrants, implying similar socioeconomic status; there might be a healthy migrant effect. In reality, rural-to-urban migrants are of lower socioeconomic status, and tend to work in jobs of much lower salaries than urban residents in China. 

### 4.3. Health Service Utilization

Our study showed that migrants were more likely to have reported chronic diseases or recent two-week morbidity than local urban residents. However, among subjects who were sick in the recent two weeks, migrants were much less likely to see a doctor as compared to local urban residents, after controlling for average monthly income, medical insurance and other variables. Our data indicated that migrants might have had significant unmet medical needs, probably due to multiple reasons. Migrants in cities might be under life pressure in finding a job, affordable housing and schools for children, and affordable health care [25]. Peng *et al*. reported that longer working hours in migrants were associated with lower probability for health care seeking behaviors [26]. Migrant workers have less disposable income for medical care than local urban residents. Yuan *et al*. found that 84.2% migrants who should see a doctor but did not thought that "there was no need to see a doctor because the feeling was not that bad". Migrant workers may be more likely to ignore and underreport illnesses [27]. Although in recent years, China has gradually established a medical insurance system allowing coverage for migrants, the medical insurance coverage is usually limited (low budget) for migrant workers, and appears not influence health seeking behaviors in migrants [28,29]. In addition, migrants have poor understanding of health care services and poor consciousness of medical insurance [30]. Reported conditions requiring hospitalization were less frequent in migrant workers *versus* local residents. It is unclear whether this might be due to more underreporting among migrant workers. Alternatively, it may be true that severe medical conditions requiring hospitalization were less frequent in migrant workers. 

## 5. Limitations

Self-reported information is prone to recall errors. It has been reported that about 5% of subjects over-reported clinic consultations over the last one month, and that self-reported health service utilization could not be taken as actual health service utilization, but reflect the overall utilization pattern [31]. We expected that such errors from self-reported data were random and would not significantly affect the validity of the comparisons. The study was carried out in factories in a rich urban area in China. Confirmative studies are required to determine whether the findings are applicable to other urban regions of China. However, migrant workers in Chinese cities do share some similarities in that they have lower socioeconomic status and often work in lower salary jobs than local urban residents, indicating that similar findings may be observed in other cities in China.

## 6. Conclusions

Chinese rural-to-urban migrant workers have lower HRQOL and higher morbidity than local urban residents, but are much less likely to see a doctor at times of illness. There is a need for improving HRQOL and access to affordable medical care for Chinese rural-to-urban migrant workers.

## List of Abbreviations

HRQOL: Health-related quality of life
PF: physical functioning
RP: Role-physical
BP: Bodily pain
GH: General health
VT: Vitality
SF: Social functioning
RE: Role-emotional
MH: Mental health
OR: Odds ratio
CI: Confidence interval
